# M-Mode and Tissue Doppler Ultrasonographic Assessment of Diaphragmatic Function in Dogs With and Without Respiratory Distress

**DOI:** 10.3390/ani15233371

**Published:** 2025-11-21

**Authors:** Jesús Talavera-López, Ariana Tur-Martín

**Affiliations:** Cardiorespiratory Service, Veterinary Teaching Hospital, Department of Animal Medicine and Surgery, Veterinary School, University of Murcia, Campus de Espinardo nº 16, 30100 Murcia, Spain

**Keywords:** diaphragmatic excursion, canine, dyspnea, ultrasound, echodiaphragmatic evaluation

## Abstract

The diaphragm plays a central role in respiration, and its dysfunction may contribute to respiratory distress (RD). Although ultrasound is widely used to assess diaphragmatic function in humans, its value in veterinary patients remains poorly defined. Fifty-eight dogs undergoing cardiorespiratory evaluation were classified as normal (*n* = 31) or with RD (*n* = 27) based on clinical scores. All underwent standardised echodiaphragmatic assessment (M-mode and tissue Doppler imaging, TDI), and interobserver measurement reproducibility was analysed. Demographic characteristics did not differ significantly between groups. Respiratory rate (RR) and body weight were the main factors influencing echodiaphragmatic parameters, with RR being the most relevant. Both groups exhibited mild asymmetry between hemidiaphragms. The mean left–right excursion difference was 4.9% (−79.9 to 79.4%) in the no-RD group and 15.2% (−63.2 to 69.6%) in RD dogs. Significant differences between groups were observed for RR (*p* = 0.039), as well as TDI-derived peak contraction velocity (*p* = 0.019) and peak relaxation velocity (*p* = 0.01). However, no single echodiaphragmatic variable demonstrated strong discriminatory ability for RD in ROC or multivariable logistic analyses. Interobserver reproducibility was excellent for all measurements (intraclass correlation coefficient > 87%). In conclusion, M-mode and TDI provide reliable quantitative evaluation of diaphragmatic motion in dogs. A mild dynamic asymmetry exists between hemidiaphragms, but RD is not necessarily indicative of diaphragmatic dysfunction. Respiratory rate remains the main factor influencing echodiaphragmatic parameters.

## Simple Summary

The diaphragm is essential for breathing, and although ultrasound is widely used to assess it in humans, its value in veterinary patients remains little known. This study explored whether ultrasound could help assess diaphragm function in dogs with breathing difficulties. The study included 58 dogs: 31 with normal breathing and 27 with an abnormal respiratory pattern. Each dog underwent a standardised echodiaphragmatic evaluation (M-mode and tissue Doppler imaging). The study also evaluated the consistency of measurements between observers. Results showed that respiratory rate, rather than diaphragm dysfunction, was the main factor affecting ultrasound measurements. Both groups of dogs displayed mild differences between the left and right sides of the diaphragm, but these differences were not linked to respiratory distress. Importantly, the ultrasound measurements demonstrated excellent inter-observer consistency. In summary, ultrasound can reliably assess diaphragm function in dogs, but respiratory distress does not necessarily indicate diaphragm problems. Instead, breathing rate has the greatest influence on the measurements.

## 1. Introduction

The diaphragm is the principal muscle responsible for respiration. Upon contraction, it exerts three primary effects: reducing intrapleural pressure, inflating the rib cage by using the abdomen as a fulcrum, and expanding the rib cage through the generation of positive intra-abdominal pressure [1]. Any disorder affecting the diaphragm alters the respiratory pattern, and, conversely, numerous respiratory diseases may compromise diaphragmatic function [2,3,4,5,6]. The most frequently reported causes of diaphragmatic dysfunction include juxtadiaphragmatic masses, diaphragmatic hernia or eventration, pneumonia, trauma, thoracic surgery, infectious and metabolic diseases, sepsis, neuropathies, myopathies, prolonged mechanical ventilation, and idiopathic etiologies [1,3,4]. The onset of diaphragmatic weakness remains uncertain; however, studies in human patients have demonstrated that diaphragmatic atrophy can develop within the first few days following the initiation of mechanical ventilation [1,2].

Fluoroscopy has historically been considered the reference standard for assessing diaphragmatic dysfunction, but it can be challenging and inaccurate, since it requires specialised equipment, radiation, and multiple operators [1]. Its dorsoventral projection limits visualisation of the posterior diaphragm, while lateral views are hindered by hemidiaphragm overlap. Because diagnosis depends on asymmetry, bilateral dysfunctions may be missed, particularly in lateral recumbency or when minimal motion, compensatory abdominal contractions, or paradoxical movements occur. Ultrasound (US) offers several benefits over fluoroscopy, including lack of ionising radiation, portable bedside approach and ease of repetition if there is a change in clinical status [1]. In human medicine, diaphragmatic US is widely employed to assess disease severity, monitor the progression of pulmonary disorders, detect phrenic nerve injury or diaphragmatic paralysis, and evaluate patients undergoing mechanical ventilation in intensive care units (ICUs), with reported high reproducibility and repeatability [2,4,5]. M-mode US has excellent temporal and axial resolution, which lends itself well to quantitative assessment of diaphragmatic motion. Diaphragmatic dysfunction can be detected by means of US based on changes in amplitude, force and velocity of contraction, special patterns of motion and changes in diaphragmatic thickness during inspiration [1,4]. The most commonly used parameters include direction of movement, amplitude of excursion—diaphragmatic excursion (DE)—and diaphragmatic thickness [1,5]. Tissue Doppler imaging (TDI), a US technique widely applied in both human and veterinary cardiology to assess myocardial function, has more recently been demonstrated to be a valuable tool for evaluating diaphragmatic function. In human ICU patients, TDI has shown promise as a predictor of successful weaning from mechanical ventilation based on changes in the TDI-derived parameters peak-contraction velocity, peak-relaxation velocity and maximal relaxation rate [7,8].

The use of M-mode US for the evaluation of the diaphragm in veterinary medicine remains poorly documented, and diaphragmatic TDI has not yet been described. In dogs, a limited number of studies have addressed the application of M-mode US to assess diaphragmatic motion. These investigations have evaluated the effects of body positioning [9,10], probe location [10], and body weight [10], as well as compared outcomes between healthy dogs and those with diaphragmatic paralysis [11]. Additionally, diaphragmatic function has been examined in dogs with cervical spinal disorders, revealing increased frequencies of dysfunction compared with control populations [12]. In cats, a recent study reported that healthy individuals exhibit significantly greater DE compared with cats affected by cardiorespiratory diseases [13]. However, reference values that clearly differentiate physiological from pathological measurements have not yet been established. Moreover, it remains unclear whether these parameters are influenced by variations in respiratory pattern.

This study aims to investigate the feasibility of M-mode and TDI for diaphragmatic assessment in dogs. By analysing quantitative echodiaphragmatic parameters, we seek to determine potential differences in diaphragmatic function between the right and left hemidiaphragms in dogs with normal respiratory patterns compared to those presenting with respiratory distress (RD). In addition, interobserver variability will be evaluated.

## 2. Materials and Methods

### 2.1. Animals

Client-owned dogs presented to the Cardiorespiratory Service of the Veterinary Teaching Hospital of the University of Murcia between September 2024 and February 2025 were considered for inclusion in this cross-sectional, prospective, observational study. Owners provided informed consent, and the Institutional Animal Care and Use Committee at the University of Murcia approved all procedures (internal code number 961/2024).

Only animals that received a complete clinical examination according to their presenting complaint and clinical signs were eligible for the study. This typically included a combination of physical examination, skull, neck and thoracic radiographs, echocardiography, pulmonary ultrasound, videofluoroscopy, airway endoscopy, computed tomography, laboratory studies (blood tests, serological studies, effusion studies, etc.) and others. The results of this examination were not included in this study, but they were used to determine the final diagnosis and posterior classification in disease groups: cardiac disease (established based on combinations of echocardiographic study, electrocardiography and chest x-ray, including congenital and acquired cardiac diseases from myocardium, valves and pericardium, independently of the presence absence of clinical symptoms of heart failure), pulmonary disease (chest x-ray, pulmonary ultrasound, computed tomography, endoscopy and bronchoalveolar lavage, including pneumonia, pulmonary fibrosis, pulmonary neoplasia, between others); upper airway disease (endoscopic examination, skull and neck x-rays/computed tomography, and fluoroscopy, including components of brachycephalic airway obstructive syndrome, pharyngeal collapse, nasopharyngeal disease, laryngeal collapse, tracheal collapse, and others); lower airway disease (chest x-ray, computed tomography, endoscopy and bronchoalveolar lavage, including bronchitis, bronchial collapse, bronchomalacia), pleural disease (chest x-ray, thoracic ultrasound, computed tomography, pleural fluid analysis, including all types of pleural effusions but cardiogenic, and intrapleural mases). Dogs not included in one of the previous categories correspond to asymptomatic dogs that underwent cardiorespiratory evaluation for different reasons (murmur investigation, blood pressure evaluation, etc.), resulting in the absence of cardiorespiratory disease.

Cases were recruited from dogs that came to the cardiorespiratory speciality consultation for evaluation, with no restriction on sex, sexual condition, weight/size or age. Demographic data were included (age, body weight, 1–9 body condition score, breed, sex, and neuter status). Dogs were excluded if they presented any condition potentially affecting the diaphragm’s structure or neuromuscular function. Exclusion criteria included diaphragmatic rupture, cervical intervertebral disc disease, prior diaphragmatic or cervical surgery, rib fractures, or intra-abdominal processes capable of causing diaphragmatic compression, such as organomegaly, pregnancy, or peritoneal effusion. Dogs were also excluded in the case of inability to obtain a clear M-mode or TDI signal of both hemidiaphragm (i.e., dogs that vocalised or exhibited aggressive behaviour).

Dogs were classified according to the respiratory pattern. A normal respiratory pattern was considered when it was quiet and barely noticeable, with the ribs moving craniolaterally and the abdomen moving slightly outward. Dogs were considered to have RD if increased ventilatory drive and/or increased work of breathing were present. The differentiation was confirmed after a thorough physical examination with special attention to the posture, mental attitude, breathing pattern (duration and effort of each respiratory phase), auscultation of the thorax and the neck, evaluation of the mucous membranes and pulses, presence of stridor or stertor, and subtle palpation of the abdominal muscles synchronically to chest auscultation and inspection, to detect effort or asynchrony. These characteristics were objectified using the clinical scoring scale included in Table 1. According to the score obtained (range 0–12), patients were classified as without RD (score 0–3) or with RD (score greater than 3 points). When present, RD was graded as mild (score 4–6), moderate (7–9), or severe (10–12).

### 2.2. Diaphragm Ultrasound Technique and Diaphragmatic Measurements

**Figure 1:**
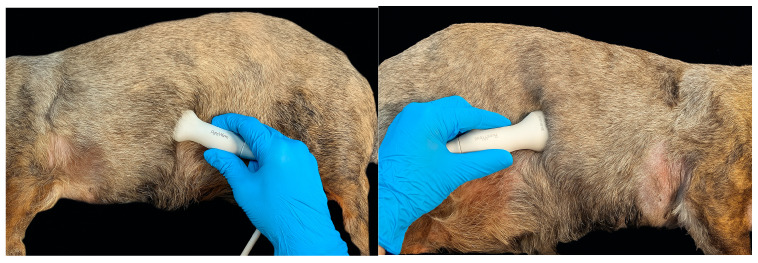
Standard positioning of the ultrasound probe for the evaluation of the left (**left image**) and right (**right image**) hemidiaphragm. The probe was angled 90–180° relative to the rib and adjusted until perpendicular to the diaphragm, then tilted to optimise visualisation of the crura.

All ultrasound studies and measurements were obtained using a Philips EPIQ Elite Diagnostic Ultrasound System (Philips Ultrasound LLC., Bothell, WA, USA: Philips Ultrasound LLC) and two ultrasound transducers with frequencies ranging from 12 to 4 MHz and 9 to 2 MHz, respectively. The animals were positioned in right and left lateral recumbence (exploration of the left and right hemidiaphragm, respectively). The transducer was positioned midway along each thoracic side, placed subcostally and directed cranially. The probe angle relative to the rib ranged from 90° to 180°, and its orientation was adjusted until the ultrasound beam was perpendicular to the diaphragm. The probe was then tilted to optimise visualisation of the diaphragmatic crura (Figure 1). The right hemidiaphragm was imaged through an acoustic window using the liver as an anatomical reference, while the left hemidiaphragm was visualised using the stomach or spleen as landmarks. To obtain accurate measurements, each dog was positioned until it was calm and relaxed.

**Figure 2:**
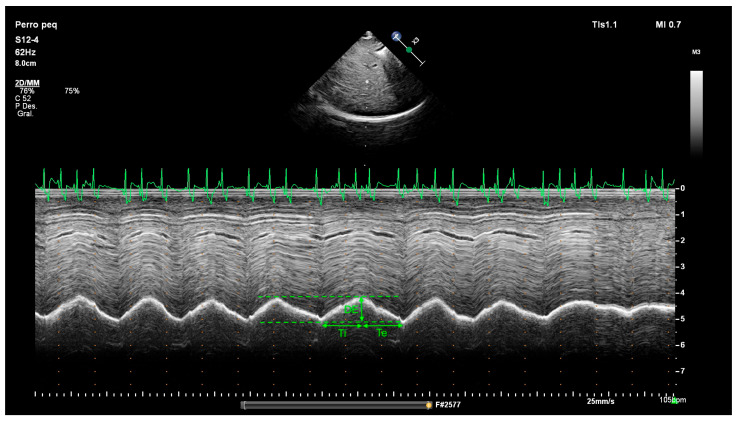
M-mode imaging in a dog showing the measurement method for diaphragmatic excursion (DE), inspiratory time (Ti), and expiratory time (Te). The contraction velocity (cm/s) can be calculated from the formula: Ve = DE/Ti. With the aid of simultaneous ECG, note the presence of sinus arrhythmia by the increase in heart rate during inspiration and the decrease during expiration.

The M-mode line was positioned perpendicular to the diaphragm to obtain the motion and amplitude excursion of the diaphragm. Under physiological conditions, diaphragmatic motion is directed toward the transducer during inspiration and reverses outward during expiration (Figure 2). On the M mode, the DE, the duration of inspiration (Ti), and the duration of expiration (Te) were measured. Respiratory rate (RR) was mathematically derived from corresponding values of Te and Ti. DE was measured from the onset of inspiration (lowest peak) to the onset of expiration (highest peak). The Ti and Te were defined as the time of up and down, respectively, of the echogenic diaphragm line. Finally, the velocity of diaphragmatic contraction (Ve) was obtained as the ratio between diaphragmatic excursion (DE) and inspiratory time (Ti).

**Figure 3:**
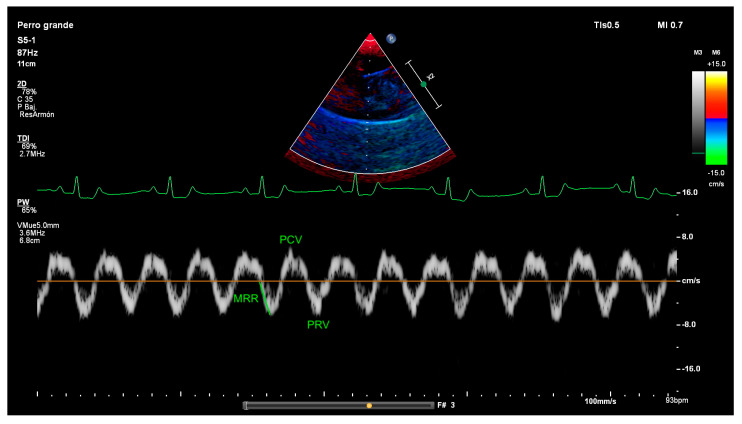
Diaphragmatic tissue Doppler imaging (TDI) in a dog showing he points where measurements were performed. Diaphragmatic TDI exhibits two waves, one during diaphragmatic contraction (above the baseline) and one during diaphragmatic relaxation (below the baseline). PCV, peak contraction velocity; PRV, peak relaxation velocity; MRV, maximal relaxation rate, and the velocity–time integral.

For TDI image acquisition, at the same M-mode position, the TDI mode was obtained by placing the cursor on the diaphragm echogenic line. As TDI measurements are angle dependent, we aimed for the ultrasound beam to reach the hemidiaphragm perpendicularly. The sample volume was adjusted to incorporate the whole range of diaphragmatic motion. On each TDI waveform, the following parameters were identified and measured (Figure 3): peak contraction velocity (PCV), peak relaxation velocity (PRV) and maximal relaxation rate (MRR). The measure of PCV and PRV was obtained by measuring the highest and the lowest peaks for inspiration and expiration, respectively. The MRR was defined as the slope of the initial steepest part of the diaphragmatic motion velocity curve during relaxation, measured in cm/s^2^.

A minimum of eight respiratory cycles was recorded for both M-mode and TDI imaging. The mean of three measurements was used as the final value for each variable. Once optimal M-mode and TDI images were obtained from each hemidiaphragm, the corresponding frozen frames were stored for subsequent offline analysis. Offline evaluations were performed blinded to patient data and group allocation. Each frozen image was graded for quality as follows: Invalid (0), uninterpretable; Low (1), at least one accurately measurable respiratory cycle; Medium (2), at least two accurately measurable cycles; and High (3), three or more accurately measurable cycles.

### 2.3. Interobserver Variability

The reproducibility of measurements performed by two different observers was assessed by randomly selecting diaphragmatic ultrasound studies (both M-mode and TDI) from 15 cases in the study. From the stored echodiaphragmatic images, two observers performed the measurements of key M-mode (DE, Ti, Te) and TDI (PCV and PRV) variables in these 15 studies at different times, without informing each other about the results. These were subsequently compared statistically (see below).

### 2.4. Statistical Analysis

A commercial software package (IBM SPSS Statistics 28.0.1.1 (14), Inc., Chicago, IL, USA) was used for statistical analysis. Statistical significance was set as *p* < 0.05. Sample size was calculated in advance with the Grammo statistical program [14]. It was therefore determined for an error probability test of 0.05, a statistical power of 0.8, and an effect size of 0.5 (medium) in 28 patients per group. The normality of each data set was analysed using the Shapiro–Wilk test. The clinical data were expressed using descriptive statistics, with results either presented as mean ± SD and 95% confidence interval (CI). The Mann–Whitney U and Wilcoxon signed-rank range tests were used for comparisons between dogs with and without RD and between hemidiaphragms, respectively. Relationships between quantitative variables were analysed by means of Spearman’s correlation. Receiver operating characteristic (ROC) curves were generated for dogs with and without RD to determine optimal cut-off values, which were identified using the Youden index. The area under the ROC curve (AUC) and corresponding 95% confidence intervals (CI) were subsequently calculated. Variables that were significantly different between dogs with and without RD were entered into a multivariate logistic regression model.

Reliability was evaluated through interobserver variability analysis, assessing absolute agreement for each variable independently. The Intraclass Correlation Coefficient (ICC) and corresponding 95% confidence intervals were calculated, with ICC values greater than 0.8 interpreted as indicating very strong agreement.

## 3. Results

### 3.1. Clinical Demographic Data

Fifty-eight dogs fulfilled the inclusion criteria and were subsequently assigned to two groups: no-RD dogs (31/58, 53.45%) and RD dogs (27/58, 46.55%). Patient demographic characteristics are presented in Table 2. At the time of enrolment, no significant differences between the two study groups in demographic variables were found (Table 2). Represented breeds included mixed breed dogs (20), Yorkshire terrier (9), Maltese (5), Chihuahua (4), English Cocker (3) Labrador Retriever (2), Poodle (2), Teckel (2) and one each of Pomeranian, American Bully, American Stanford, Beagle, Boxer, Cavalier King Charles Spaniel, Irish Wolfhound, Lhassa Apso, German Sheperd, Belgian Sheperd, and W West Highland White Terrier.

In the group of RD dogs, a significantly higher percentage of dogs had mild distress (13/27, 48.1%), followed by moderate distress (9/27, 33.3%) and a lower percentage had severe distress (5/27, 18.5%).

With respect to the underlying disease condition (Table 2), most of the patients of both groups (with and without RD) presented cardiac disease (31/58, 53.4%). In the case of the group without RD and cardiac disease (19/31, 61.3%), these correspond to dogs in the asymptomatic phase of chronic valvular heart disease (16/19, 84.2%), mild dilated cardiomyopathy phenotype (1/19, 5.3%), mild subaortic stenosis (1/19, 5.3%) and mild pulmonary hypertension (1/19, 5.3%). Three and one dog in the no-RD group presented, respectively, upper (dynamic pharyngeal collapse) and lower (chronic bronchitis) respiratory disease, but without inducing pathological changes in respiratory pattern at the moment of inclusion. The other asymptomatic dogs in the no-RD group (8/31, 25.8%) corresponded to patients without cardiorespiratory disease. This included five dogs with ophthalmologic disease that were referred for blood pressure evaluation (within range) and three dogs with innocent murmurs (echocardiography). The distribution of the dogs with RD in function of underlying disease also showed a predominance of dogs with cardiac disease (12/27, 44.4%), including dogs with chronic valvular heart disease (9/12, 75%) and dogs with pulmonary hypertension (3/12, 25%). Pulmonary diseases diagnosed in the group of dogs with RD (7/27, 25.9%) were pneumonia (5/7, 71.4%) and neoplasia (2/7, 28.6%). Upper airway diseases inducing RD in this group (5/27, 18.5%) were brachycephalic airway obstructive syndrome (3/5, 60%) and pharyngeal collapse (2/5, 40%). One dog presented with RD because of bronchial collapse (lower airway disease group) and two more secondary to neoplastic pleural disease (pleural disease group).

### 3.2. M-Mode Results

High-quality M-mode images could be obtained in 52/58 (89.7%) dogs from the left hemidiaphragm and in 50/58 (86.2%) from the right hemidiaphragm. Medium-quality images were obtained in 5/58 (8.6%) dogs from left and right hemidiaphragms. In one dog (1/58, 1.7%), the M-mode images could not be measured from his left hemidiaphragm and in three dogs (3/58, 5.2%), from their right hemidiaphragm. Thus, the mean ± SD of M-mode quality (0–3 scale) was 2.92 ± 0.28 for the left hemidiaphragm and 2.91 ± 0.28 for the right hemidiaphragm.

The M-mode diaphragmatic parameters of the study dogs are presented in Table 3. Significant differences in DE between right and left hemidiaphragms were found for all dogs and for dogs with RD (*p* = 0.008 and *p* = 0.048, respectively). The mean difference between the left and right DE was 9.7% (−79.9 to 79.4%). In 18/53 (34%) dogs, the DE from the right diaphragm was lower than from the left and vice versa in 35 dogs (66%).

When comparing results between dogs with and without RD, significant differences were found in Ti, Te, and RR (Table 3). There was a significant correlation between most M-mode parameters and RR, mainly when all dogs or only dogs without RD were considered. Body weight demonstrated correlations with several parameters, most notably when all dogs were considered in the analysis. Correlation coefficients and associated *p*-values are summarised in Table 4. The mean difference between the left and right DE was 4.9% (−79.9 to 79.4%) in the no-RD group and 15.2% (−63.2 to 69.6%) in the RD group. In 10/29 (34.5%) dogs of the no-RD group and in 8/25 dogs (32%) of the RD group, the DE from the right diaphragm was lower than from the left and vice versa in the rest. These differences were higher than 50% in 7/29 no-RD dogs (24.1%) and in 4/25 RD-dogs (16%).

ROC analysis was performed to assess the ability of M-mode parameters to distinguish between dogs with and without RD and to determine potential cut-off values. The results indicated that none of the parameters demonstrated sufficient accuracy to reliably differentiate between the two groups. The best result corresponds to RR obtained from the right hemidiaphragm (AUC 0.660, *p* = 0.019, sensitivity 68%, specificity 77%, cut-off value 35.18 rpm) (Table 5). In the multivariable logistic analysis that included the M-mode variables showing significant differences between RD and no-RD dogs in the univariate analysis, none of them showed a significant association with the presence of RD (Table 6).

### 3.3. Tissue Doppler Results

High-quality TDI images could be obtained in 27/58 (46.5%) dogs from the left hemidiaphragm and in 30/58 (51.7%) from the right hemidiaphragm. Medium-quality images were obtained in 16/58 (27.6%) dogs from left and 14/58 (24.1%) from right hemidiaphragms. Low quality was attributed to the TDI images of 16/58 (27.6%) dogs from left and 8/58 (13.8%) from right hemidiaphragms. In three dogs (3/58, 5.2%), the TDI images were invalid to be measured from their left hemidiaphragm and in six dogs (6/58, 10.4%), from their right hemidiaphragm. Thus, the mean ± SD of TDI quality (0–3 scale) was 2.28 ± 0.80 for the left hemidiaphragm and 2.44 ± 0.74 for the right hemidiaphragm.

The TDI diaphragmatic parameters of the study dogs are presented in Table 3. Significant differences in PRV between right and left hemidiaphragms were found for all dogs and RD dogs (*p* = 0.012 and *p* = 0.033, respectively) and on PCV for no-RD dogs (*p* = 0.011). When comparing results between dogs with and without RD, significant differences were found in PCV and PRV obtained from the left hemidiaphragm (Table 3). There was a significant correlation between most TDI parameters and RR, mainly when all dogs or no-RD dogs were considered. Body weight was only correlated with some parameters, mainly when all dogs were included in the analysis. Table 4 summarises the correlation coefficients and *p*-values.

The ROC analysis was used to evaluate the ability of TDI parameters to differentiate the cut-off value between the RD and no-RD groups. The result indicated that any parameter shows enough accuracy to discriminate between these groups. The best result corresponds to PRV obtained from the left hemidiaphragm (AUC 0.702, *p* = 0.005, sensitivity 54%, specificity 83%, cut-off value 5.16 cm/s) (Table 5). In the multivariable logistic analysis that included the TDI variables showing significant differences between dogs with and without RD in the univariate analysis, none of them showed a significant association with the presence of RD (Table 6).

### 3.4. Interobserver Variability

Excellent interobserver reproducibility was found for all tested variables, both M-mode and TDI derived, with an ICC above 87% for all measurements, a mean ICC of 96.7%, and a *p*-value < 0.001 (Table 7).

## 4. Discussion

To the knowledge of the authors, this study is the first to explore the use of TDI for assessing diaphragmatic function in dogs. It also presents the first comparative analysis of M-mode and TDI parameters in dogs with and without RD, providing novel quantitative data derived from ultrasonographic assessment of the diaphragm. The present results indicate that the protocol applied in this study enables the acquisition of high-quality images suitable for quantitative assessment. A mild functional asymmetry between hemidiaphragms was observed, and the presence of RD did not appear to reflect diaphragmatic dysfunction. Instead, respiratory rate emerged as the main factor influencing echodiaphragmatic parameters.

Diaphragmatic paralysis has been described in several animal species [15,16,17,18,19,20,21] with a wide range of associated respiratory signs, although it is more widely described in humans [22]. Some patients are asymptomatic, and it is only found incidentally (unilateral), but others (usually bilateral paralysis) may manifest orthopnea or dyspnea during exertion to ventilatory failure, followed by cyanosis with apnea [3,11,22]. Considering the critical contribution of the diaphragm to respiratory dynamics, diaphragmatic dysfunction could also be a contributing factor to the presence of RD, not exclusively in cases of diaphragmatic paralysis, but also accompanying other diseases causing respiratory compromise, as has been described in human patients with acute heart failure [23]. The DE values obtained in the present study are similar to those of previous studies in healthy dogs [10,11]. When comparing DE values between dogs with and without RD, no significant differences were obtained. These findings suggest that diaphragmatic function adapts appropriately to the pathological condition and is unlikely to play a central role in the respiratory pattern alterations observed in the dogs of this study. In human ICU patients, a special predisposition to muscle degradation exists, with the diaphragm being particularly susceptible due to its thin structure and rapid atrophy compared to skeletal muscles [1,5,6]. In no-ICU patients, unilateral or bilateral diaphragmatic paralysis is caused by insufficiency of the phrenic nerve in adverse neuromuscular conditions (e.g., trauma, compression or degeneration of the phrenic nerve, and myopathy) and other conditions (e.g., pneumonia and idiopathic conditions). Further studies in veterinary patients undergoing prolonged intubation in ICU units are warranted to determine whether serial diaphragmatic echocardiographic assessment could help guide weaning and adjust the prognosis of these patients.

The assessment of diaphragmatic dysfunction generally relies on a combination of clinical evaluation, pulmonary function testing (such as spirometry, phrenic nerve stimulation, and electromyography), together with imaging modalities including radiography, fluoroscopy, computed tomography (CT), magnetic resonance imaging (MRI), and ultrasonography (US) [24,25]. Among these approaches, US is particularly advantageous due to its accessibility, absence of ionising radiation, cost-effectiveness, and high reproducibility [1,26,27]. Ultrasonography also enables both qualitative and quantitative assessment of diaphragmatic morphology and motion, providing valuable information on muscle thickness and contractile behaviour. As in humans [1,27,28], diaphragmatic US has been previously validated as a good method to evaluate DE and other parameters in dogs [10,11] and cats [13]. Our findings align with previous studies and confirm that diaphragmatic US is a relatively simple and objective method for measuring diaphragmatic movement in dogs, offering excellent repeatability between operators. Previous studies have focused on comparing healthy dogs with dogs with diaphragmatic paralysis [11] or cervical spinal disorders [12], as well as between healthy and diseased cats [13]. However, this is the first study comparing dogs with and without RD. This study was designed under the assumption that diaphragmatic dysfunction is unlikely in individuals with a normal respiratory pattern. From this perspective, and considering the results as a whole, it can be inferred that diaphragmatic function was largely preserved—though with some variability—in dogs exhibiting abnormal respiratory patterns associated with common cardiorespiratory conditions. However, it is important to note that the dogs with normal breathing patterns in this study were not strictly healthy. They were dogs with subclinical heart disease, innocent murmurs, or eye conditions. Although these conditions are unlikely to have influenced the echodiaphragmatic parameters, this possibility cannot be fully excluded. Moreover, the dogs with RD in this study presented diseases of heterogeneous aetiology, which may have limited the identification of alterations specific to a given respiratory disorder or compensatory mechanism. Future studies including healthy dogs selected through standardised criteria, as well as stratified analyses by disease type and respiratory pattern, are warranted to confirm and extend the present findings.

Mild diaphragmatic asymmetry is considered a normal variant in dogs under radiographic [29] and fluoroscopic evaluation [3]. Previous echodiaphragmatic studies in healthy dogs and cats have shown a non-significant asymmetry in DE favourable to the left diaphragm, with a mean left-right difference of 20% [10,11,13]. Contrarily, in dogs with unilateral diaphragmatic paralysis, the left side had lower M-mode DE values than the right, with a difference of 55% [11]. The dogs in the present study also showed asymmetrical function based on M-mode DE, although the differences were significant only in the RD group. Thus, the right DE in this group was significantly greater than that of the left. Furthermore, the ratio of the DE between both hemidiaphragms was greater in the group of dogs with RD (15.2% vs. 4.9% in no-RD dogs). These results could indicate that in dogs with RD, even without a significant reduction in DE, functional asymmetry could be accentuated. In any case, considering an isolated individual, this asymmetry is not key in the appearance of RD since it was very pronounced (more than 50%) in more non-RD dogs (7/29, 24.1%) than in RD dogs (4/25, 16%).

Doppler ultrasonography operates by detecting frequency shifts produced by sound waves reflected from moving structures. TDI, an echocardiographic modality based on the same physical principles, quantifies the low-velocity, high-amplitude signals generated by myocardial motion. TDI provides reproducible and easily obtainable measurements that have proven useful in a range of cardiac disorders. Nevertheless, its application to the evaluation of diaphragmatic motion has been scarcely investigated in humans [7,8,30] and, to date, has not been reported in dogs. Human studies have found TDI parameters to help predict successful weaning of ICU patients on mechanical ventilation (a known cause of diaphragmatic dysfunction) [7,8], with improved accuracy when combined with M-mode DE values [8]. The findings of this study offer novel information on diaphragmatic motion velocity patterns and reference ranges in dogs with normal respiration and in those exhibiting RD. The TDI profile observed was consistent with the mild functional asymmetry detected in M-mode DE values. Notably, PCV and PRV of the left hemidiaphragm were significantly higher in dogs with RD compared to dogs with normal respiratory patterns, suggesting a possible compensatory mechanism. Overall, ROC analysis revealed low sensitivity and specificity of both M-mode and TDI echodiaphragmatic parameters in distinguishing normal from dyspneic breathing patterns. However, the highest AUC values were observed for TDI-derived parameters. Collectively, these findings suggest that a combined assessment of TDI parameters and DE may be clinically relevant and potentially prognostic in veterinary patients. Further studies with larger cohorts and longitudinal designs are therefore recommended to confirm these observations.

Several intrinsic factors may influence echodiaphragmatic parameters. Previous studies in dogs have reported a significant correlation between M-mode DE and body weight when a wide weight range was included in the sample [10]. However, this association was not observed when only dogs of similar body weight (e.g., beagles) were evaluated, nor in cats, in which the adult weight range is comparatively narrow [13]. In the present study, the impact of body weight on multiple parameters was also evident when all 58 dogs were analysed together, but the effect was attenuated—both in the number of affected parameters and in its magnitude—when each group was considered separately (Table 4). Importantly, no significant differences in body weight were detected between groups (Table 2). These findings suggest that body weight may influence echodiaphragmatic measurements, particularly in animals at the extremes of the weight spectrum, and should therefore be taken into account during clinical evaluation.

Respiratory rate is a physiological variable closely linked to respiratory function and is regulated to meet the metabolic demands of oxygen delivery and carbon dioxide elimination [31]. Both alterations in RR and modifications of the respiratory pattern represent key mechanisms for maintaining adequate respiratory function, with hypoxia, hypercapnia, and acidosis serving as potent stimulatory drivers of ventilation [31]. In the present study, RR had a significant impact on most echodiaphragmatic parameters, both when considering the entire cohort of 58 dogs and when analysing dogs with and without RD separately. Furthermore, RR was significantly higher in the group of dogs with RD. Although this influence is physiologically expected, it has not been assessed in previous veterinary studies, in which RR was not analysed as a variable [3,11,12,13]. Diagnostic studies performed in humans with suspected diaphragmatic dysfunction use conscious forced respirations [1,26,30] or are performed in ICU patients undergoing mechanical ventilation [4,5,6,8]. In these circumstances, RR is a controllable factor. However, in a veterinary clinical context, a wide range of RR is expected, influenced by both stress and potential diseases. Thus, its influence must be considered in clinical assessments of diaphragmatic function in dogs.

While this study is the first to compare diaphragmatic function using US in dogs with and without RD and to describe diaphragmatic TDI in dogs, several limitations must be acknowledged. These include the relatively small sample size, single-centre data collection, and potential operator variability. The range of underlying diseases causing RD is heterogeneous, which may have made it difficult to find differences attributable to a particular aetiology or breathing strategy. Multi-centre investigations employing standardised protocols will be essential to validate and expand upon these findings. No objective method has been used to determine the degree of oxygenation of dogs. However, the main aim of the study was to determine if changes in the breathing pattern could be associated with possible diaphragmatic dysfunction, assessed by ultrasound, and not to determine the severity of hypoxemia or establish a correlation with the onset of distress or changes in breathing pattern. On the other hand, it is well known that pulse oximetry has significant limitations affecting the technique itself, leading to overestimates of desaturation. Furthermore, that is accentuated when applied to conscious, non-sedated/non-anaesthetised animals, as readings can be significantly affected by hair or hyperpigmentation. Regarding other techniques, such as arterial blood gas analysis, these provide the best accuracy but require skill and technique, and samples are not always easy to obtain in patients without sedation. Furthermore, it would have made it more difficult to recruit candidates for the study, which was designed to be non-invasive. In contrast, this study has used a grading system using scores attributed to the main characteristics that determine the presence/absence of a normal breathing pattern in a thorough physical examination of patients, whereas in many studies, the determination of the presence of DR is merely subjective [32,33]. In addition, the cross-sectional design did not allow for assessment of longitudinal changes in diaphragmatic motion. Future studies investigating diaphragmatic function as a dynamic parameter could provide valuable insights, particularly regarding early diagnosis and prognostic evaluation.

## 5. Conclusions

This study provides the first comparative analysis of echodiaphragmatic parameters in dogs with and without RD, as well as the first description of diaphragmatic TDI in this species. The protocol applied yielded high-quality images suitable for quantitative assessment. The quantitative data presented herein may serve as a reference for future experimental and clinical evaluations. A mild degree of functional asymmetry between hemidiaphragms was identified. Importantly, the presence of RD did not necessarily indicate diaphragmatic dysfunction; rather, respiratory rate emerged as the primary factor influencing echodiaphragmatic parameters.

## Figures and Tables

**Table 1:** Clinical scoring system used to assess the presence or absence of respiratory distress.

Clinical Parameter (Resting Dogs in Consultation Room)	Score
Behaviour attitude towards breathing	Relaxed, no explicit breathing attention = 0 Glazed expression, anxious breathing = 1
Body posture	Comfortable, natural = 0 Persistent standing or sitting = 1 Stretched neck, nasal flare, orthopnoea = 2
Rib movement	Subtle craniolateral movement = 0 Rib marking, intercostal spaces sink = 1
Abdominal movement	Slightly outward = 0 Accentuated, in opposition to the costal movement = 1
Activation of the abdominal muscles during exhalation (abdominal palpation)	No activation is perceived = 0 Active and prolonged contraction of abdominal muscles during exhalation = 1
Respiratory rhythmicity	Short and compensated respiratory phases = 0 Prolonged inhalation/exhalation = 1
Synchronization of thoracic and abdominal movement	Synchronous = 0 Asynchronous = 1
Perception of respiratory effort	Smooth and barely noticeable = 0 Difficult or laboured breathing = 1
Tolerance to breathing with the mouth closed (either spontaneous or manually closed)	Complete = 0 It causes discomfort and manifests as effort = 1
Presence of stridor, crackles, snoring (pre-auscultation)	Missing = 0 Present = 1
Wheezing or crackles (pulmonary auscultation)	Missing = 0 Present = 1

Interpretation of the scale (range 0–12 points): 0–3, no respiratory distress; 4–12, respiratory distress.

**Table 2:** Patient Baseline Characteristics and Between-Group Comparison.

	All *n* (%)	Respiratory Distress *n* (%)		*p*-Value (No vs. Yes)
		No	Yes	
	58 (100)	31 (53.4)	27 (46.6)	
Age and size ^				
Age (years)	10.64 ± 3.94 (9.6–11.67)	10.65 ± 3.99 (9.18–12.11)	10.63 ± 3.96 (9.06–12.19)	0.802
Body weight (kg)	7.50 ± 10.03 (8.50–13.77)	9.67 ± 7.88 (6.78–12.56)	12.82 ± 11.98 (8.08–17.56)	0.322
Body condition (1–9 scale)	5.43 ± 1.34 (5.08–5.78)	5.42 ± 1.02 (5.04–5.8)	5.44 ± 1.65 (4.79–6.10)	0.417
Sex				
Male	33 (56.9)	18 (58.1)	15 (55.6)	0.847
Female	25 (43.1)	13 (41.9)	12 (44.4)	
*p*-Value (column)	0.294	0.369	0.564	
Sexual Condition				
Intact males	18 (31)	9 (29)	9 (33.3)	0.501
Neutered males	15 (25.9)	9 (29)	6 (22.2)	
Intact females	13 (22.4)	5 (16.1)	8 (29.6)	
Neutered females	12 (20.7)	8 (25.8)	4 (14.8)	
*p*-Value (column)	0.694	0.709	0.535	
Grade of respiratory distress				
No distress	31 (53.5)	31 (100)	-	**<0.001**
Mild	13 (22.41)	-	13 (48.1)	
Moderate	9 (15.5)	-	9 (33.3)	
Severe	5 (8.6)	-	5 (18.5)	
*p*-Value (column)	**<0.001**	-	**0.017**	
Disease group				
Asymptomatic	8 (13.8)	8 (25.8)	-	**0.002**
Cardiac disease	31 (53.4)	19 (61.3)	12 (44.4)	
Pulmonary disease	7 (12.1)	-	7 (25.9)	
Upper airway disease	8 (13.8)	3 (9.7)	5 (18.5)	
Lower airway disease	2 (3.4)	1 (3.2)	1 (3.7)	
Pleural disease	2 (3.4)	-	2 (7.4)	
*p*-Value (column)	**<0.001**	**<0.001**	**0.006**	

^ Data are the mean ± SD (95% confidence interval); *p*-Values in bold: statistically significant differences *(p* < 0.05).

**Table 3:** Echo-derived Diaphragmatic parameters and Between-Group Comparisons.

	Hemi-Diaphragm	All	Respiratory Distress		*p*-Value (No vs. Yes)
			No	Yes	
M-mode					
DE (mm)	Right	8.89 ± 4.40 (7.70–10.08)	9.06 ± 4.85 (7.25–10.87)	8.69 ± 3.89 (7.09–10.30)	0.548
	Left	7.31 ± 3.76 (6.32–8.31)	7.41 ± 3.27 (6.19–8.63)	7.21 ± 4.31 (5.5–8.91)	0.472
*p* -Value (column)		**0.008**	0.074	**0.048**	
Inspiration time (ms)	Right	785.69 ± 478.91 (656.22–915.15)	885.50 ± 472.72 (708.98–1062.02)	665.91 ± 467.57 (472.91–858.91)	**0.033**
	Left	712.44 ± 377.14 (612.37–812.51)	814.87 ± 372.13 (675.91–853.82)	598.63 ± 355.45 (458.02–739.24)	**0.031**
*p* -Value (column)		0.094	0.214	0.209	
Expiration time (ms)	Right	977.36 ± 674.30 (795.08–1159.65)	1127.27 ± 763.05 (842.34–1412.19)	797.48 ± 507.62 (587.94–1007.02)	0.080
	Left	878.72 ± 605.16 (718.15–1039.29)	1026.30 ± 682.68 (771.38–1281.22)	714.74 ± 464.66 (530.93–898.55)	**0.048**
*p* -Value (column)		0.137	0.245	0.288	
Ve (cm/s)	Right	13.74 ± 9.98 (11.04–16.44)	12.12 ± 7.20 (9.43–14.81)	15.69 ± 12.42 (10.56–20.82)	0.151
	Left	12.30 ± 7.83 (10.21–14.40)	10.44 ± 5.35 (8.45–12.44)	14.45 ± 9.63 (10.57–18.34)	0.097
*p* -Value (column)		0.080	0.218	0.209	
RR (rpm)	Right	53.15 ± 46.63 (40.55–65.76)	47.01 ± 51.82 (27.66–66.36)	60.53 ± 39.28 (44.32–76.65)	**0.043**
	Left	58.17 ± 45.91 (45.99–70.35)	46.37 ± 36.27 (32.82–59.91)	71.28 ± 52.28 (50.60–91.96)	**0.039**
*p* -Value (column)		0.116	0.538	0.115	
Tissue Doppler					
PCV (cm/s)	Right	4.30 ± 2.06 (3.73–4.87)	4.20 ± 1.68 (3.53–4.86)	4.41 ± 2.44 (3.41–5.42)	0.855
	Left	4.05 ± 1.92 (3.53–4.57)	3.53 ± 1.37 (3.01–4.05)	4.63 ± 2.28 (3.71–5.56)	**0.019**
*p* -Value (column)		0.052	**0.011**	0.797	
PRV (cm/s)	Right	5.44 ± 3.08 (4.58–6.29)	4.87 ± 2.17 (4.01–5.73)	6.04 ± 3.78 (4.48–7.60)	0.268
	Left	4.64 ± 2.32 (4.02–5.27)	3.88 ± 1.37 (3.36–4.40)	5.49 ± 2.85 (4.34–6.64)	**0.010**
*p* -Value (column)		**0.012**	**0.033**	0.179	
MRR (cm/s^2^)	Right	61.39 ± 58.86 (43.91–78.87)	45.36 ± 32.72 (30.85–59.87)	76.09 ± 73.00 (45.26–106.91)	0.141
	Left	56.97 ± 36.80 (46.29–67.66)	46.71 ± 26.34 (35.59–57.83)	67.23 ± 43.05 (49.05–85.41)	0.124
*p* -Value (column)		0.871	0.421	0.390	

Data are the mean ± SD (95% confidence interval), *p*-Value Yes–No: Mann–Whitney U test; *p*-Value (column): Wilcoxon signed-rank test. DE, diaphragmatic excursion; RR, M-mode-derived respiratory rate; Ve, contraction velocity; PCV, peak contraction velocity; PRV, peak relaxation velocity; MRR, maximal relaxation rate; *p*-Values in bold: statistically significant differences (*p* < 0.05).

**Table 4:** Two-tailed Spearman Correlations between body weight and selected M-mode and Tissue Doppler-derived variables.

	BW	RRe	R-DE	L-DE	R-Ve	L-Ve	R-PCV	L-PCV	R-PRV	L-PRV
All dogs										
BW		0.194	0.442 **	0.287 *	0.591 **	0.432 **	0.242	0.248	0.350 *	0.326 *
RRe	0.194		−0.305 *	−0.490 **	0.454 **	0.472 **	0.505 **	0.477 **	0.421 **	0.408 **
R-DE	0.442 **	−0.305 *		0.608 **	0.387 **	0.177	0.143	0.121	0.269 *	0.321 **
L-DE	0.287 *	−0.490 **	0.608 **		0.117	0.383 **	0.128	0.108	0.192	0.211
R-Ve	0.591 **	0.454 **	0.387 **	0.117		0.612 **	0.619 **	0.410 **	0.606 **	0.514 **
L-Ve	0.432 **	0.472 **	0.177	0.383 **	0.612 **		0.558 **	0.500 **	0.630 **	0.565 **
R-PCV	0.242	0.505 **	0.143	0.128	0.619 **	0.558 **		0.511 **	0.795 **	0.684 **
L-PCV	0.248	0.477 **	0.121	0.108	0.410 **	0.500 **	0.511 **		0.447 **	0.735 **
R-PRV	0.350 *	0.421 **	0.269 *	0.192	0.606 **	0.630 **	0.795 **	0.447 **		0.722 **
L-PRV	0.326 *	0.408 **	0.321 **	0.211	0.514 **	0.565 **	0.684 **	0.735 **	0.722 **	
Dogs with normal respiratory pattern										
BW		0.110	0.645 **	0.321	0.639 **	0.306	0.144	0.211	0.229	0.277
RRe	0.110		−0.284	−0.495 **	0.398 *	0.543 **	0.544 **	0.495 **	0.493 *	0.403 *
R-DE	0.645 **	−0.284		0.564 **	0.514 **	0.065	0.098	0.025	0.195	0.228
L-DE	0.321	−0.495 **	0.564 **		0.143	0.346	−0.101	−0.010	0.091	0.036
R-Ve	0.639 **	0.398 *	0.514 **	0.143		0.542 **	0.520 **	0.377 *	0.558 **	0.481 **
L-Ve	0.306	0.543 **	0.065	0.346	0.542 **		0.433 *	0.369	0.621 **	0.321
R-PCV	0.144	0.544 **	0.098	−0.101	0.520 **	0.433 *		0.548 **	0.734 **	0.637 **
L-PCV	0.211	0.495 **	0.025	−0.010	0.377 *	0.369	0.548 **		0.339	0.708 **
R-PRV	0.229	0.493 *	0.195	0.091	0.558 **	0.621 **	0.734 **	0.339		0.389 *
L-PRV	0.277	0.403 *	0.228	0.036	0.481 **	0.321	0.637 **	0.708 **	0.389 *	
Dogs with respiratory distress										
BW		0.237	0.204	0.258	0.440 *	0.467 *	0.323	0.112	0.435 *	0.314
RRe	0.237		−0.376	−0.549 **	0.476 *	0.393 *	0.503 *	0.396 *	0.338	0.330
R-DE	0.204	−0.376		0.778 **	0.297	0.326	0.127	0.187	0.288	0.398
L-DE	0.258	−0.549 **	0.778 **		0.183	0.475 *	0.160	0.149	0.315	0.371
R-Ve	0.440 *	0.476 *	0.297	0.183		0.695 **	0.732 **	0.358	0.654 **	0.545 **
L-Ve	0.467 *	0.393 *	0.326	0.475 *	0.695 **		0.710 **	0.524 **	0.647 **	0.671 **
R-PCV	0.323	0.503 *	0.127	0.160	0.732 **	0.710 **		0.398	0.805 **	0.691 **
L-PCV	0.112	0.396 *	0.187	0.149	0.358	0.524 **	0.398		0.363	0.685 **
R-PRV	0.435 *	0.338	0.288	0.315	0.654 **	0.647 **	0.805 **	0.363		0.813 **
L-PRV	0.314	0.330	0.398	0.371	0.545 **	0.671 **	0.691 **	0.685 **	0.813 **	

** The correlation is significant at the 0.01 level (two-tailed). * The correlation is significant at the 0.05 level (two-tailed). DE, diaphragmatic excursion; RRe, M-mode-derived respiratory rate; Ve, contraction velocity; PCV, peak contraction velocity; PRV, peak relaxation velocity; L-, left hemidiaphragm; R-, right hemidiaphragm.

**Table 5:** Results of the ROC analysis to evaluate the ability of M-mode and Tissue Doppler parameters to differentiate the presence of respiratory distress.

	Hemi-Diaphragm	AUC	*p*-Value	Youden Index	Cut-Off Value	Sensibility	Specificity
M-mode							
DE	Right	0.486	0.859	0.100	2.73	1.0	0.1
	Left	0.444	0.482	0.122	11.16	0.22	0.9
	Both	0.469	0.575	0.100	12.19	0.25	0.85
Ti	Right	0.332	0.027	0.027	190.5	0.96	0.07
	Left	**0.346**	**0.037**	0	1702	0	1
	Both	**0.349**	**0.004**	0.017	147	1.0	0.02
Te	Right	0.362	0.074	0.067	147	1.0	0.07
	Left	**0.348**	**0.037**	0	3624	0	1
	All	**0.358**	**0.007**	0.017	113.5	1.0	0.07
Ve	Right	0.613	0.137	0.293	9.82	0.76	0.53
	Left	0.629	0.085	0.246	16.55	0.35	0.90
	Both	**0.624**	**0.019**	0.256	9.66	0.71	0.55
RR	Right	**0.660**	**0.037**	0.347	35.18	0.68	0.77
	Left	**0.659**	**0.029**	0.278	61.89	0.44	0.83
	Both	**0.659**	**0.002**	0.304	37.62	0.65	0.65
Tissue Doppler							
PCV	Right	0.515	0.856	0.133	2.94	0.80	0.33
	Left	**0.685**	**0.012**	0.358	4.41	0.46	0.90
	Both	0.600	0.068	0.211	3.12	0.77	0.45
PRV	Right	0.590	0.259	0.200	9.25	0.20	1.0
	Left	**0.702**	**0.005**	0.366	5.16	0.54	0.83
	Both	0.644	0.007	0.240	5.34	0.49	0.75
MRR	Right	0.627	0.127	0.250	31.80	0.75	0.50
	Left	0.629	0.111	0.292	55.65	0.54	0.75
	Both	**0.629**	**0.024**	0.197	22.70	0.96	0.24

Bold: The pair AUC-*p*-Value is statistically significant (*p* < 0.05). DE, diaphragmatic excursion; Ti, inspiration time; Te, expiration time; Ve, contraction velocity; RR, respiratory rate; PCV, peak contraction velocity; PRV, peak relaxation velocity; MRR, maximal relaxation rate.

**Table 6:** Final model from multivariate logistic regression analysis of variables associated with respiratory distress.

	Odds Ratio (95% CI)	*p*-Value
BW	0.996 (0.925–1.073)	0.921
RRe	0.979 (0.941–1.019)	0.304
R-Ti	1.0 (0.997–1.003)	0.998
L-Ti	0.998 (0.993–1.003)	0.470
L-Te	1.0 (0.998–1.001)	0.678
L-PCV	1.109 (0.630–1.952)	0.721
L-PRV	1.576 (0.902–2.756)	0.110

BW, body weight; RRe, M-mode-derived respiratory rate; Ti, inspiratory time; Te, expiratory time; PCV, peak contraction velocity; PRV, peak relaxation velocity; L-, left hemidiaphragm; R-, right hemidiaphragm.

**Table 7:** Interobserver variability.

	Hemi-Diaphragm	ICC	95% CI	*p*-Value
M-mode				
DE	Right	0.956	0.862–0.986	<0.001
	Left	0.886	0.662–0.962	<0.001
Ti	Right	0.989	0.966–0.996	<0.001
	Left	0.976	0.927–0.992	<0.001
Te	Right	0.987	0.960–0.996	<0.001
	Left	0.981	0.943–0.994	<0.001
Tissue Doppler				
PCV	Right	0.970	0.906–0.990	<0.001
	Left	0.952	0.856–0.984	<0.001
PRV	Right	0.996	0.986–0.999	<0.001
	Left	0.972	0.917–0.991	<0.001

ICC, intraclass coefficient; CI, confidence interval; DE, diaphragmatic excursion; Ti, inspiration time; Te, expiration time; PCV, peak contraction velocity; PRV, peak relaxation velocity.

## Data Availability

The original contributions presented in this study are included in the article. Further inquiries can be directed to the corresponding author.

## Abbreviations

The following abbreviations are used in this manuscript:

AUC	Area under the curve
CI	Confidence interval
DE	Diaphragmatic excursion
ICC	Intraclass correlation coefficient
ICU	Intensive care unit
MRR	Maximal relaxation rate
PCV	Peak contraction velocity
PRV	Peak relaxation velocity
RD	Respiratory distress
ROC	Receiver operating characteristic
RR	Respiratory rate
TDI	Tissue Doppler Imaging
Te	Expiratory time
Ti	Inspiratory time
US	Ultrasound
Ve	Diaphragmatic contraction velocity
